# Host‐specific soil microbes contribute to habitat restriction of closely related oaks (*Quercus* spp.)

**DOI:** 10.1002/ece3.9614

**Published:** 2022-12-12

**Authors:** Yingtong Wu, Alicia Brown, Robert E. Ricklefs

**Affiliations:** ^1^ Department of Biology University of Missouri–St. Louis St. Louis Missouri USA; ^2^ Whitney R. Harris World Ecology Center University of Missouri–St. Louis St. Louis Missouri USA

**Keywords:** habitat distributions, habitat divergence, host specificity, plant–soil (below‐ground) interactions, *Quercus*, soil microbes

## Abstract

Habitat divergence among close relatives is a common phenomenon. Studying the mechanisms behind habitat divergence is fundamental to understanding niche partitioning, species diversification, and other evolutionary processes. Recent studies found that soil microbes regulate the abundance and diversity of plant species. However, it remains unclear whether soil microbes can affect the habitat distributions of plants and drive habitat divergence. To fill in this knowledge gap, we investigated whether soil microbes might restrict habitat distributions of closely related oaks (*Quercus* spp.) in eastern North America. We performed a soil inoculum experiment using two pairs of sister species (i.e., the most closely related species) that show habitat divergence: *Quercus alba* (local species) vs. *Q. michauxii* (foreign), and *Q. shumardii* (local) vs. *Q. acerifolia* (foreign). To test whether host‐specific soil microbes are responsible for habitat restriction, we investigated the impact of local sister live soil (containing soil microbes associated with local sister species) on the survival and growth of local and foreign species. Second, to test whether habitat‐specific soil microbes are responsible for habitat restriction, we examined the effect of local habitat live soil (containing soil microbes within local sister's habitats, but not directly associated with local sister species) on the seedlings of local and foreign species. We found that local sister live soil decreased the survival and biomass of foreign species' seedlings while increasing those of local species, suggesting that host‐specific soil microbes could potentially mediate habitat exclusion. In contrast, local habitat live soil did not differentially affect the survival or biomass of the local vs. foreign species. Our study indicates that soil microbes associated with one sister species can suppress the recruitment of the other host species, contributing to the habitat partitioning of close relatives. Considering the complex interactions with soil microbes is essential for understanding the habitat distributions of closely related plants.

## Graphical abstract

Why is a species only found in a certain habitat but not in others? Why do closely related species show divergence in habitat preference? Our findings show that host‐specific soil microbes, not habitat‐specific soil microbes, contribute to habitat restriction and partitioning among closely related species.

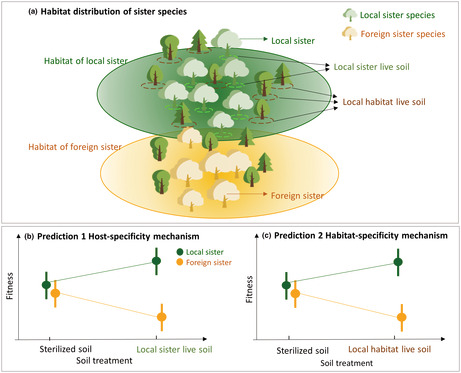

## 1 INTRODUCTION

Understanding the mechanisms underlying species habitat distributions has been a long‐standing issue in ecology, biogeography, and evolution (Bazzaz, 1991; MacArthur, 1972; Rabinowitz, 1981; Sexton et al., 2017). Habitat differentiation among closely related species is frequently observed in a wide range of taxa, especially in species‐rich clades, such as monkeyflowers (*Mimulus*), oaks (*Quercus*), and silver‐sword (*Argyroxiphium*, *Dubautia*, and *Wilkesia*; Blonder et al., 2015; Cavender‐Bares et al., 2004; Sobel, 2014). Traditionally and intuitively, researchers associate abiotic variables, such as resource levels, microclimates, soil conditions, and light intensity, to divergent habitat distributions among close relatives. On the other hand, biotic interactions can also limit geographic distributions of a host species. Particularly, the roles of seed predators, herbivores, and soil microbes on species distributions is an active area of research (Alexandre et al., 2018; Benning & Moeller, 2020; Gaston, 2009; McCarthy‐Neumann & Ibáñez, 2012). However, most of these studies have not linked these biotic interactions among close relatives with habitat restriction, that is, limited occurrence of a species to certain habitat(s) within its geographic range. More empirical evidence is needed to answer the question: how do biotic interactions restrict habitat distributions and promote habitat partitioning among closely related species?

Recent research has found that biotic interactions can mediate habitat exclusion among closely related plant species. For example, in multiple plant taxa in Amazonian rainforests, herbivores drive clay‐soil specialist plants to occur only in clay‐soil forests because of their low tolerance to herbivory in white‐sand forests, while their close relatives, white‐sand specialists, withstand the intensive herbivory better and remain occupying white‐sand forests (Fine et al., 2004, 2013). Consistently, numerous studies have found that herbivores limit plant distributions by restricting hosts to a smaller subset of habitats within their physiological tolerance, and consequently, the specialization to marginal habitats helps the disadvantaged host escape from intensive herbivory that otherwise they would have encountered in the primary habitat of the other host (Benning et al., 2019; Parker & Root, 1981; Pizano et al., 2011; Rand, 2003).

The roles of soil microbes in regulating plant species abundance and diversity are coming to the surface in recent years (Comita et al., 2010; LaManna et al., 2017; Marden et al., 2017). Theoretically, microbial communities can also mediate mutual exclusion of habitats and range distributions of plants (Bever et al., 2015; Holt & Bonsall, 2017), given that they can be transmitted and infect among closely related hosts in a similar fashion as generalist herbivores. Most examples supporting this hypothesis involve the introduction of exotic species that are carriers of novel pathogens, which decrease populations of native close relatives (Engelkes et al., 2008; Paillet, 2002; Tompkins et al., 2000, 2003). One textbook example is that of the introduced Japanese Chestnut (*Castanea crenata*), which transmitted a canker fungus, *Cryphonectria parasitica*, and devastated populations of the native American Chestnut (*Castanea dentata*) in eastern North America (Rhoades & Park, 2001). Limited evidence suggests that soil microbes from native species can constrain distributions of native close relatives as well. For instance, range‐restricted plant species typically are more susceptible to soil negative feedback when grown in the live soil from closely related species, while widespread species are much less affected by this feedback from native close relatives (Kempel et al., 2018; Liu et al., 2012). These results suggest that habitat specialists might be suppressed by soil microbes from the widespread congeneric relatives. Other studies found that habitat segregation among closely related species is caused by local adaptation to arbuscular mycorrhizal fungi found in their own soil habitats: transplanted ecotypes/species show poorer performance due to maladaptation to the fungal communities in a novel habitat, making them less competitive compared to the local host (Osborne et al., 2018; Pizano et al., 2011). In addition, positive soil feedback to conspecific seedlings (Bennett et al., 2017; McCarthy‐Neumann & Ibáñez, 2012) indicates that host‐specific microbes can promote and maintain the dominance of conspecific individuals in its native habitat over heterospecific individuals, which drives habitat exclusion among close relatives. Particularly, tree species associated with ectomycorrhizal fungi often show positive conspecific soil feedback (Bennett et al., 2017). While hinted, these studies have not directly tested whether and how soil microbes contribute to habitat restriction among closely related hosts.

To reveal the potential roles of soil microbes in habitat restriction, two distinctive mechanisms should be considered. The first mechanism is that soil microbes associated with one host plant exclude the other host species from invading its habitat. This mechanism assumes that the composition and functions of soil microbes are host‐specific, even among closely related plants. Indeed, a host species effect on soil microbial composition has been found in congeneric species (Morris et al., 2008, 2009). Additionally, this mechanism suggests that soil microbes associated with one species might be harmless or beneficial to the coevolved host, while they are parasites or insufficient mutualists to the novel host. This mechanism counters the well‐known Janzen–Cornell Hypothesis, which posits that soil microbes promote coexistence of heterospecific individuals through conspecific negative density dependence (Connell, 1971; Janzen, 1970). Indeed, recent synthesis indicates that Janzen–Cornell Hypothesis sensu stricto is often applies only to tropical ecosystems and to species without strong association to mutualistic fungi; for temperate ecosystems and for species strongly associated with mutualistic fungi, Janzen–Cornell Hypothesis is often violated and positive conspecific soil feedback is expected (Comita et al., 2010; Hyatt et al., 2003; LaManna et al., 2017; Liang et al., 2015; McGuire, 2007).

The second mechanism is that soil microbes associated with the local habitat of one host species might exclude the other host from expanding to the new habitat. This mechanism assumes that soil microbial communities are habitat‐specific, and that host plants are negatively affected by cross‐habitat soil microbes. Supporting this assumption, previous literature reports that soil microbial communities vary with habitat types (Wang et al., 2021; Yang et al., 2016); additionally, transplanted host plants are negatively affected by soil microbes of novel habitats (Osborne et al., 2018; Pizano et al., 2011). While the first mechanism emphasizes host‐specific composition and function of soil microbes, the second one emphasizes habitat specificity. These two mechanisms are not mutually exclusive, but we need to test them separately to identify which pathway contributed to habitat divergence.

**FIGURE 1:**
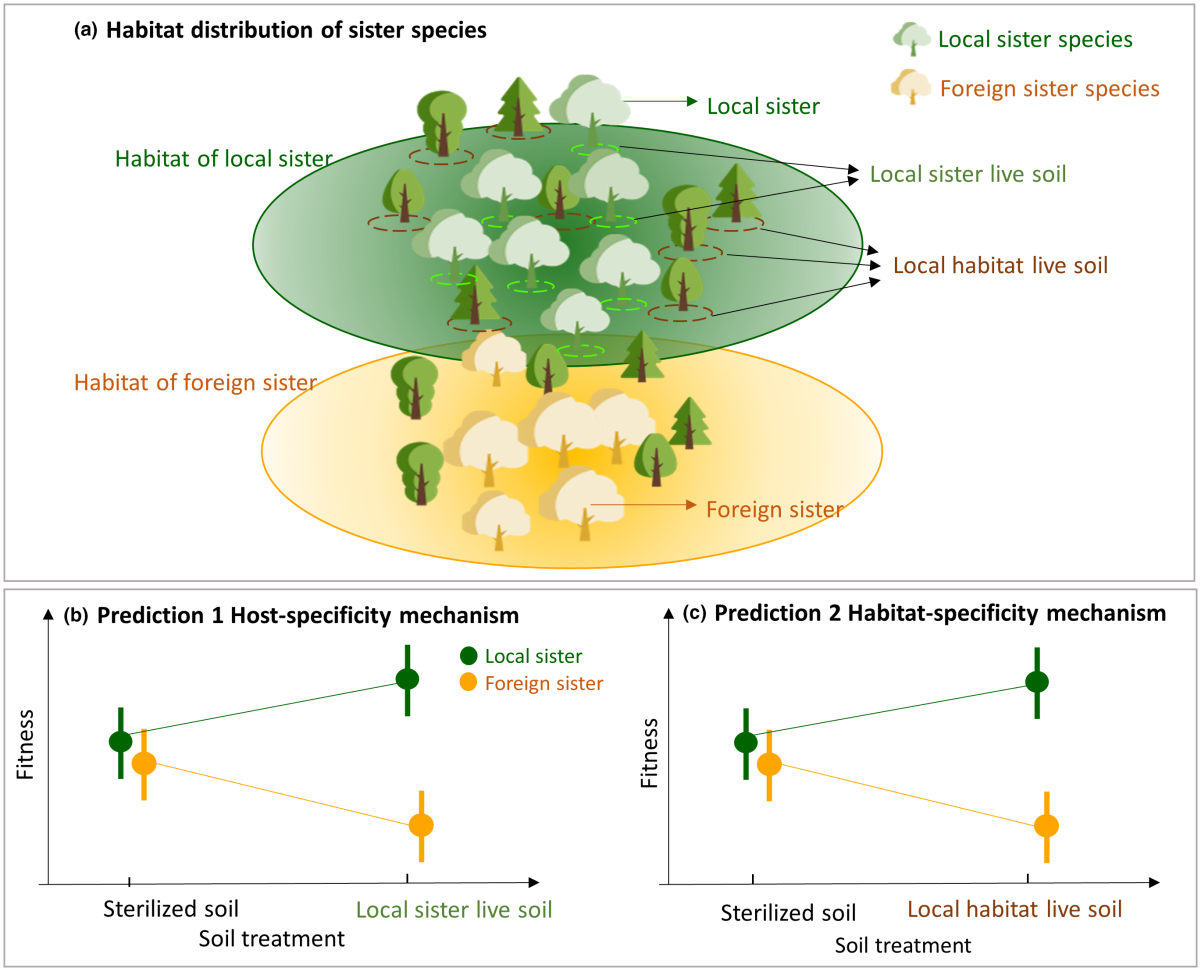
Hypothesis of soil microbe‐mediated habitat restriction of sister species. This diagram visualizes the predictions that soil microbes of a local sister constrain habitat distribution of its foreign sister species. (b) Prediction 1—host‐specificity mechanism: local sister live soil collected from adult trees of local sister species (green dashed circles in panel a) increases the fitness of conspecific seedlings due to specialized soil mutualists and tolerance of its own pathogens, while the same soil decreases the fitness of foreign sister's seedlings due to soil pathogens parasitic to the foreign sister and foreign sister's susceptibility. (c) Prediction 2—habitat‐specificity mechanism: local habitat live soil collected from other species co‐occurring within local sister's habitat (brown dashed circles in panel a) differentially affects the fitness of local sister's and foreign sister's seedlings.

We suggest that these two mechanisms can be separately tested using habitat‐divergent sister species in a soil inoculum experiment, as explained below (Figure 1). If soil microbes directly associated with sister species limit habitat distribution (the host‐specificity mechanism), one would predict that live soil associated with one sister species (Figure 1; hereafter “local sister live soil”) should decrease the fitness of foreign sister's seedlings from a different habitat. This is because soil pathogens from the local sister can be parasitic to the foreign sister, and/or foreign sister is inherently more susceptible to local sister's pathogens. Alternatively, local sister live soil might have no effects on the foreign sister species, acting as insufficient mutualists. In contrast, we expect that live soil associated with local sister should support higher fitness of its own seedlings due to specialized soil mutualists and higher tolerance of local sister to its own pathogens (Figure 1b; Prediction 1). Thus, we would expect a strong interaction effect between host habitat origin (local sister vs. foreign sister) and the soil treatments (local sister live soil vs. sterilized soil). Sterilization of local soil would cancel both of these effects. A lack of interaction effect, or an interaction effect opposite to the predicted direction, would lead us to reject the host‐specificity mechanism. Similarly, we can test the habitat‐specificity effect (Figure 1c; Prediction 2): if cross‐habitat soil microbes constrain habitat distribution, one would predict that general microbes from local sister's native habitat (which are not directly associated with the roots of local species, hereafter “local habitat live soil”) should decrease the fitness of foreign sister's seedlings, while increasing the fitness of the local sister species. By experimenting with two different types of live soils, namely local sister live soil and local habitat live soil, we can distinguish the contributions of these two mechanisms in maintaining habitat partitioning of host plants.

In this study, we used two sister‐species pairs of oaks (*Quercus* spp.) in a soil inoculum experiment to test the potential role of soil microbes in restricting species habitats. We found evidence that habitat restriction could potentially be microbially mediated. By showing how local biotic interactions affect population dynamics, this study has practical implications for planning conservation of native habitat specialists (DeCesare et al., 2009; Flores‐Tolentino et al., 2020; Recart et al., 2012).

## 2 METHODS

### 2.1 Study system

We used two oak sister‐species pairs (*Q. alba*‐*Q. michauxii*, *Q. shumardii*‐*Q. acerifolia*) in the soil inoculum experiment (Figure S1). In the sister pair *Q. alba*‐*Q. michauxii*, *Q. alba* grows on dry upland slopes to well‐drained loam and is widely distributed throughout the eastern U.S., while *Q. michauxii* is adapted to wet bottomlands and is abundant in the southeastern U.S. (Stein et al., 2003). While *Q. alba* and *Q. michauxii* have overlapping species ranges, our field observations and an analysis confirm that the two species occupy separate habitats and rarely co‐occur in close proximity within the same site (see Note S1). In the sister pair *Q. shumardii*‐*Q. acerifolia*, *Q. shumardii* is restricted to well‐drained soils along streams and rivers and is widely distributed in the southeastern U.S. (Stein et al., 2003). In contrast, *Q. acerifolia* is adapted to xeric soils on mountain ridges and occurs at only four known locations where *Q. shumardii* has not been found in close proximity (pers. obs. by the first author and communications with knowledgeable local botanists; Figure S1d). A recent genomic analysis by Hipp et al. (2018) confirmed their sister–species relationships. Hereafter, we use the term “foreign sister” for *Q. michauxii* and *Q. acerifolia*, in relation to our experimental sites within or close to St. Louis, MO (38.64°N, 90.24°W), which are beyond the natural habitats of these two species (Figure S1). In contrast, we use the term “local sister” for *Q. alba*, *Q. shumardii*.

Oak species encounter many taxa of soil pathogens, including soil fungi (Balci et al., 2007; Haavik et al., 2015; Rizzo et al., 2002), root‐parasitic nematodes (Maboreke et al., 2016), and ectomycorrhizal fungi that occasionally turn parasitic depending on external environments and host species (Ibáñez & McCarthy‐Neumann, 2016; Johnson et al., 1997; Nash et al., 2020). Despite the high diversity of soil pathogens, previous research found positive conspecific soil feedback in oaks (Bennett et al., 2017; McCarthy‐Neumann & Ibáñez, 2012). Specifically, oaks are predominantly associated with ectomycorrhizal fungi (Soudzilovskaia et al., 2020), a group of fungi that often generates positive conspecific soil feedback and facilitates the recruitment of conspecific individuals (Bennett et al., 2017). Our Prediction 1 that seedlings of local sister grow better in conspecific live soil (Figure 1b) is derived from these previous findings in oaks. For our study species, we did not directly test the underlying assumption that soil microbes can be transmitted among sister species, but this assumption is probably true because phylogenetically related host plants share similar root‐associated pathogens (Liu et al., 2012; Schroeder et al., 2019). In oaks, a portion of ectomycorrhizal fungi can also be shared among co‐occurring species while the rest can be host‐specific (Aponte et al., 2010; Morris et al., 2009). The roles of these shared ectomycorrhizal fungi can be host‐dependent: they might be beneficial to the co‐evolved local host, but are harmful or have no effects on the novel host.

### 2.2 Acorn collection

Acorns were collected from early October to early November 2018 from the Shaw Nature Reserve (Gray Summit, MO; 38.48°N, 90.82°W), the Missouri Botanical Garden (St. Louis, MO; 38.61°N, 90.26°W), and the campus of University of Missouri–St. Louis (St. Louis, MO; 38.71°N, 90.31°W), depending on the availability of each species' acorns at each location. Specifically, for foreign sister species, we collected acorns from two mature trees of *Q. michauxii* in the Missouri Botanical Garden, and from one mature tree of *Q. acerifolia* in the Shaw Nature Reserve (see Note S2 for provenance). For local sister species, we collected acorns from two trees per species. To ensure that seed source and maternal effects (Fort et al., 2021) did not confound the treatment effect, we used the same seed source composition for each treatment within the same species.

We selected healthy acorns by visually inspecting and excluding acorns with damages, and then used float tests to further exclude floating acorns that are nonviable (Morina et al., 2017); only the healthy “sinkers” were kept and stored in bags with moist and sterilized sphagnum moss at 4°C for stratification. All seeds were stratified until early April 2019, when most acorns showed radicals. We only used acorns with radicals for the experiment, since acorns that did not show radicals were likely nonviable.

### 2.3 Soil inoculum experiment

We set up a soil inoculum experiment in a climate‐controlled greenhouse at the University of Missouri–St. Louis from April to August 2019. Deep tree pots (10.16 cm diameter, 35.56 cm depth) were cleaned carefully using 10% bleach before the experiment. We used commercial soil (Berger BM7 35% Bark HP; Berger Company) for the background soil, which made up 90% of the soil in all the pots; this ensured that the nutrition levels and soil structure in all pots were consistent. This background soil was sterilized in an autoclave twice with a 24‐h interval, at 121°C for 75 min each time; double sterilization prevents growth of any heat‐resistant strains.

Two types of live soil were collected from two natural forests: the Shaw Nature Reserve and the Tyson Research Center (Eureka, MO; 38.53°N, 90.56°W), in late March 2019. The first type of live soil was associated with the mother trees of local sister (corresponding to green dashed circles in Figure 1a), representing the local sister live soil. We collected the live soil from the bases of two mature trees from each of the local species, *Q. alba* and *Q. shumardii*, from three locations within the Shaw Nature Reserve. We collected the soil in cores of 20 cm depth and 10 cm radius, at three points 1–1.5 m distant from the tree trunk. Thus, local sister live soil consisted of live soil from three trees for each sister pair. The live soils were mixed within the host species to allow maximum statistical power in the experiment, especially when sampling intensity of soils is low in our study (Cahill Jr et al., 2017). While we are aware of the debate regarding issues of soil sample pooling (Reinhart & Rinella, 2016; Rinella & Reinhart, 2019), a recent meta‐analysis found no evidence that soil sample pooling systematically biases estimates of plant–soil feedback direction, magnitude, or variance (Allen et al., 2021).

The second type of inoculum was live soil containing general microbes that the foreign oak species have not encountered, whereas the local sister have encountered in their own habitats (corresponding to the brown dashed circles in Figure 1a), representing the local habitat live soil. This live soil was randomly collected from 10 locations within 1–1.5 m from the base of other tree species (listed in Note S3) within the Tyson Research Center. We drove down a service road and stopped at distances (m) corresponding to 10 randomly drawn numbers; at each stop, we collected soil from at least 30 m off the road under the appropriate host trees. The samples were then combined into a soil mixture. Other than our study species, the most dominant tree species at the Tyson Research Center are *Q. rubra* (relative abundance 21.61%) and *Q. velutina* (10.59%). The nearest distance to a neighboring tree is 3.53 m on average, and 20% of individuals have a nearest distance = 2.0 m, and 40% with a nearest distance = 2.8 m. Thus, our sampling included a certain extent of soil microbes interspaced among trees.

We set up four soil treatments in the greenhouse. (1) Sterilized soil, which included 10% sterilized general local soil in addition to the 90% sterilized background soil. (2) Local sister live soil (green circles in Figure 1a), which included 10% live soil from the mother trees of the local species *Q. alba* (for pots containing seeds of *Q. alba* and *Q. michauxii*), or from the mother trees of the other local species *Q. shumardii* (for pots containing seeds of *Q. shumardii* and *Q. acerifolia*). (3) Local habitat live soil (brown circles in Figure 1a), which included 10% local habitat live soil collected from the base of other host plants. (4) Local habitat live soil plus fungicide treatment, which had the same soil mixture as treatment (3), to which we applied Ridomil Gold MZ WG fungicide (Syngenta Crop Protection) on the soil surface every 2 weeks following manufacturers' instructions. This fungicide, generally used to eliminate soil pathogens, has reportedly limited effects on ectomycorrhizal fungi (Bell et al., 2006; Maron et al., 2011; Norghauer et al., 2010). We applied this fungicide to examine whether the elimination of soil pathogens from local habitat live soil had an impact on the seedlings; specifically, if we found a significant increase in performance of foreign sister under treatment (4) compared to soil treatment (3), it would suggest that soil pathogens from local habitat live soil can effectively suppress foreign sister, lending support to Prediction 2. Note that this study did not investigate the effect of the foreign sister's microbes on the local sister seedlings, nor did we include the treatment of local sister live soil plus fungicide, due to our limited efforts on soil collection and greenhouse work. Regardless, the treatments used in this study met the minimum requirements needed to test the two separate mechanisms (Figure 1).

Live soils were added to the pots within 4 days after field collection. These soil mixtures were manually homogenized before potting. To minimize soil splashing across pots, we filled soils only to 30.5 cm deep for all tree pots (35.56 cm‐deep pots). Each soil treatment mentioned above had 10 replicates (pots) per species, resulting in a total of 160 pots in the greenhouse. In each pot, one viable acorn was planted immediately beneath the soil surface. Seed source, seed length, and seed width were documented for each pot to statistically control for potential effects of mother tree and seed size on seedling survival and growth (Bonfil, 1998; Shi et al., 2019). In our experiment, seed size was not differentiated among soil treatments nor host habitat origin (*p* > .80); thus, it should not confound the main effect of soil treatments or habitat origin. Pots were randomly distributed within the greenhouse so that spatial variation of environmental variables did not confound experimental results. Pots were spaced at least 15 cm apart to minimize cross‐over of soil microbes. We watered the pots every 5–6 days with a water hose serving one pot at a time to avoid soil splashing. A shade cloth with 40% light penetration was hung in the greenhouse to mimic the light environment within natural forests.

Seedling survival, height, diameter of the widest aboveground part, and leaf number were recorded at the end of August 2019, after 150 days since the initial planting. Previous studies show that the survival curve of oak seedlings levels off after 80 days (Badano et al., 2009; González‐Salvatierra et al., 2013), and thus, our time span of 150 days allowed us to capture the most critical stage of development. We also harvested surviving seedlings to measure the aboveground biomass and belowground biomass. Aboveground biomass was measured as the seedling dry weight above the emergence point from the acorn. Roots were carefully separated from soil and were washed to remove all attached soil particles. Belowground biomass was measured as the dry weight of the roots. Total biomass was the sum of the above and belowground biomass.

### 2.4 Data analyses

To test the effects of soil microbes on seedling survival and growth, we first fitted full models for separate response variable using maximum‐likelihood models as implemented in the R package *lme4* (Bates et al., 2014): we used (1) a generalized linear mixed model (GLMM) with a binomial distribution for survival rate, (2) linear mixed‐effect models (LME) for the total, aboveground, belowground biomass as well as seedling height and diameter, and (3) a GLMM with a Poisson distribution for leaf number. Seedling biomass and height were log‐transformed to meet the requirement of a normal distribution. For each model, we first defined the full model and then performed model selection. In the full model, we included soil treatments, host habitat origin (local vs. foreign sister), and their interaction term as the fixed‐effect factors. We also included seed length and seed width as fixed‐effect factors to account for possible effects of seed size, because previous research shows that seed size is positively related to oak seedling survival and growth (Aizen & Patterson III, 1990; Aizen & Woodcock, 1996; Quero et al., 2007). Species identity and species pairs were also included as fixed‐effect factors, instead of random‐effect factors because they only have two levels (Crawley, 2002). Mother tree was included as a random‐effect factor. We then used “dredge” function from the R package *MuMIn* (Barton, 2010) to generate a set of models with combinations of fixed‐effect terms from the full model, and used the corrected Akaike Information Criterion (AICc) to identify the best model (Table S1). Since testing our hypotheses requires testing the significance of the interaction term between soil treatment and host habitat origin (as illustrated in Figure 1b,c), we kept soil treatments, host habitat origin, and their interaction term during model selection.

After identifying the best model (Table S1), we then obtained distribution of each parameter within a Bayesian framework with Markov chain Monte Carlo (MCMC) in Stan as implemented in the R package *rstanarm* (Goodrich et al., 2018). Specifically, we used the “stan_glmer” functions for generalized linear mixed‐effect model, or “stan_lmer” functions for linear mixed‐effect model. This Bayesian inference method is a simulation technique to obtain the distribution of each parameter in a model (Zuur & Ieno, 2016), which is suited for the small sample size in our study. We focused on interpreting the Bayesian inference also because the maximum‐likelihood models mentioned above, implemented in package *lme4*, occasionally reported singular fits due to small sample size. We set the model prior as a Cauchy distribution with center 0 and scale 2.5 for each model, which is a weakly informative prior recommended by (Gelman et al., 2008). Each model ran for 2000 iterations (1000 “burn‐in” iterations followed by 1000 sample iterations) in each of four chains. We used the default “adept_delta” (target average proposal acceptance probability) = 0.95 during Stan's adaptation period, or when necessary, we increased it to 0.99 until no divergent transitions were detected. Model convergence of the Bayesian models was evaluated by examining *Rhat* (the ratio of between‐chain variance to within‐chain variance) and the effective number of simulation draws (Gelman & Rubin, 1992). Statistical significance of the effects is indicated when 90% credible interval (CIs) or 80% CIs of the Bayesian point estimates do not include zero. Using the 90% CIs is a conservative threshold, while using the 80% CIs is a slightly more liberal threshold (Gomes et al., 2021). When significant interaction term was detected, results were visualized using the estimated marginal means of the best Bayesian model, which was implemented with the “emmeans” function in the R package *emmeans* (Lenth et al., 2019). All statistical analyses were performed in R version 3.5.0 (R Core Team, 2018).

## 3 RESULTS

**FIGURE 2:**
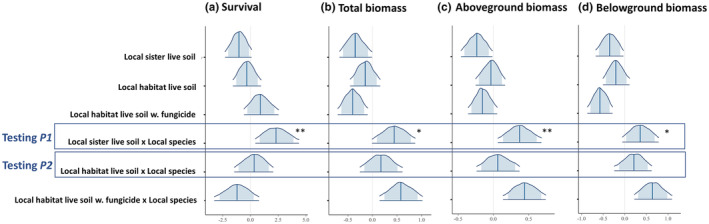
Bayesian estimates of the effects of soil treatments and host habitat origin (local species vs. foreign species) on oak seedling survival and biomass in a soil inoculum experiment. Sterilized soil is used as a reference level for soil treatment, and foreign species is used as a reference level for host habitat origin. Blue vertical lines represent median estimates of the coefficients derived from the Bayesian models. The truncated distribution outline represents 90% credible intervals (CIs), while the shaded‐light blue region represents 80% CIs. A light‐gray vertical line marks *x* = 0 in each panel. The tests for Prediction 1 ( *P1* ) and Prediction 2 ( *P2* ) are highlighted with rectangles. Statistical significance is highlighted with asterisks: ** indicates that 90% CIs of the posterior estimates of the coefficient do not overlap with zero, while * indicates that the 80% CIs do not include zero.

**FIGURE 3:**
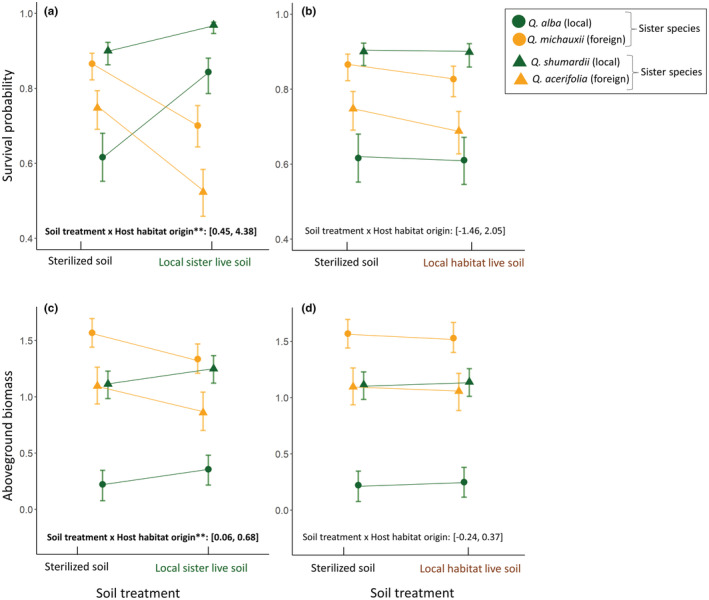
Seedling survival probability and aboveground biomass of the local vs. foreign sister species in different soil treatments. Values were derived from the best Bayesian model, using estimated marginal means. Panels (a, c) compare the survival probabilities and aboveground biomass of local sister (green points) when grown in sterilized soil vs. in local sister live soil, and the survival of foreign sister (yellow points) in these two treatments. Panels (b, d) compare the survival probabilities and aboveground biomass of local sister (green points) when grown in sterilized soil vs. in local habitat live soil that does not associate specifically with one host, and the survival of foreign sister (yellow points) in these two treatments. Error bars represent one standard error. Statistical significance, as tested using Bayesian models, is highlighted with asterisks: ** indicates that 90% credible intervals of the posterior distribution of the model coefficient do not overlap with zero. The 90% credible intervals are marked on each panel.

The results for greenhouse seedling survival were consistent with Prediction 1, that is, local sister live soil reduced survivorship of the foreign sister species, but not of the local sister (Figures 2 and 3). The final model includes soil treatment, host habitat origin, and species identity as fixed variables, and mother tree identity as random variable (Table S1). Consistent with Prediction 1 (Figure 1b), we detected a significant interaction between host habitat origin and the treatment of local sister live soil in the direction that we predicted (90% CI does not overlap zero; Figures 2a and 3a, Table S2). The results were consistent for both species pairs (*Q. alba*‐*Q. michauxii*, and *Q. shumardii*‐*Q. acerifolia*). Specifically, when planted in the soil inoculated with conspecific species' live soil, seedlings of the local sister survived better than in sterilized soil, while seedlings of the foreign sister survived less well in local sister live soil than in sterilized soil (Figure 3a). Contrary to Prediction 2 (Figure 1c), we did not find significant interaction effect between host habitat origin and soil treatment of local habitat live soil on seedling survival (Figures 2a and 3b, Table S2).

The results of the greenhouse experiment for seedling biomass were also consistent with Prediction 1, and again held for both species pairs (Figures 2 and 3). When planted in the soil inoculated with the local sister live soil, seedlings of the foreign sister had significantly lower aboveground biomass compared to seedlings of the local sister (90% CI of the interaction term does not overlap zero; Figures 2c and 3c, Table S3). Inconsistent with Prediction 2, soil inoculation with local habitat live soil did not differentially impact the aboveground biomass for local sister vs. foreign sister, as compared to the sterilized soil (Figure 3d, Table S3). Results for total biomass and belowground biomass were similar to that of aboveground biomass (Figure 2b,d; Figure S2).

For seedling height, diameter, and number of leaves, we did not detect a significant interaction between host habitat origin and soil treatment of local sister live soil (Figure S3; Table S4). Seed size was positively related to seedling biomass, height, diameter, and number of leaves (Table S4).

When comparing the effects of the fungicide treatment vs. no fungicide in local habitat live soil, we did not find a significant increase in performance of foreign sister under the fungicide treatment, indicating that soil pathogens from local habitat live soil did not suppress seedlings of the foreign sister (Tables S2–S4). This is inconsistent with our Prediction 2. Rather, the fungicide treatment increased the aboveground biomass and seedling diameter of only local sister (Tables S3 and S4).

## 4 DISCUSSION

While abiotic conditions have been considered the main drivers of species distributions, recent research has increasingly emphasized the roles of biotic interactions in mediating plant performance and species distributions (Pigot & Tobias, 2013, reviewed by Wisz et al., 2013). We used a carefully designed experiment to investigate whether and how soil microbes could contribute to limiting species habitat distributions in an ecologically dominant and diverse clade—oaks (*Quercus* spp.) in North America. We identified and tested two separate mechanisms through which soil microbes could restrict host habitat: the first mechanism is that sister species have host‐specific soil microbes that can inhibit the growth and survival of the other sister species; the second mechanism is that sister species are adapted to habitat‐specific soil microbes, and perform poorly when encountering soil microbes from novel habitats.

We found that host‐specific soil microbes (the first mechanism), but not habitat‐specific microbes (the second mechanism), contribute to habitat restriction of sister species. Specifically, when seedlings of foreign sister species (*Q. michauxii*, *Q. acerifolia*) grew in the live soil of the local sister (*Q. alba*, *Q. shumardii*), the probability of survival and biomass decreased compared to when growing in sterilized soil (Figure 3a,c); in contrast, local sister species did not show decreased survival or reduced biomass when growing in their own live soil, but increased performance, compared to growing in sterilized soil. This suggests that soil microbes associated with one sister species can inhibit the other sister species from occupying the habitat by decreasing seedling survival and growth. In other words, our experiment shows that host‐specific soil microbes can promote habitat partitioning between the hosts.

Previous studies have found that plant–soil interactions can limit species distributions. For instance, when the annual plant *Clarkia xantiana* ssp. *xantiana* was transplanted beyond its habitat, soil microbes decreased lifetime fitness of the transplanted individuals while the home‐range live soil improved the fitness (Benning & Moeller, 2020). Other transplant experiments also found survival of the transplanted species to be restricted by the presence of soil fungal pathogens or the absence of soil mutualists (Brown & Vellend, 2014; Carteron et al., 2020). Notably, our result differs from these previous experiments that tested maladaptation to the general microbes beyond the range or habitats of the transplanted host; in those studies, the live soil inoculum was not associated with sister species or close relatives of the target host. In fact, our experiment indicated that general soil microbes beyond the foreign sister's habitats did not suppress the survival or growth of the seedlings (Figure 3b,d), suggesting that maladaptation to general microbes of novel habitats does not restrict habitat distributions of our study species. Our application of a pathogen‐targeted fungicide on local habitat live soil did not improve the seedling survival or growth of foreign sister (Tables S2–S4), further confirming that habitat‐specific soil pathogens did not explain habitat restriction of foreign sister species. Instead, we found that host‐specific soil microbes explained their poor performance when growing in the soil microbial environments of their sister species (Figure 3a,c). This could be because that habitat‐specific microbes collected from nonsister species are less effective in transmitting to the foreign species, given that phylogenetical relatedness of host species correlates positively with the proportion of shared microbes (Liu et al., 2012; Schroeder et al., 2019).

Consistent with our finding and Prediction 1, Kempel et al. (2018) found that soil microbes from widespread and possibly habitat‐generalist hosts more strongly suppressed the growth of the regionally rare close relatives than their widespread relatives. The same pattern was found in Amazonian plants: herbivores specific to a forest type prevent confamilial relatives from coexisting together within the same forest habitat (Fine et al., 2004). Uniquely, our study shows that the mosaic co‐existence of close relatives through niche partitioning, or a checkerboard pattern of close relatives, can be produced through the effects of shared biotic interactions belowground. While we acknowledge that species habitat distributions are often determined predominantly by inherent environmental tolerance, rather than by biotic interactions (Manthey et al., 2011), the effects of soil microbes on host plants can be perceived as extended phenotypes of the hosts. Our findings support the concept that plant habitat distributions are affected by their responses to specific fungi groups (Afkhami et al., 2014; Gerz et al., 2018; Singh et al., 2011).

Several mechanisms might explain the effects of host‐specific microbes on habitat restriction, as observed in our study. First, different host plants co‐evolve with, and adapt to, their local pathogens, and when sister species come into contact, transmission of novel pathogens can reduce the fitness of the foreign sister species (Petipas et al., 2021). Second, the lack of microbial mutualists in novel soil habitats might assist pathogen invasion by allowing faster transmission rates. Specifically, ectomycorrhizal fungi are host‐specific soil mutualists in oaks (Aponte et al., 2010; Morris et al., 2009), and the association with beneficial ectomycorrhizal fungi assists host defense against root pathogens (Mohan et al., 2015; Vishwanathan et al., 2020). Without the protection of host‐specific ectomycorrhizal fungi, pathogens transmitted from close relatives might invade faster into the roots of the foreign sister species. Third, from a genetic perspective, genes related to disease resistance (R‐genes) might lead to specialized recognition of, and defense against, only a small subset of pathogens (Marden et al., 2017). Maintaining multiple defense pathways is likely costly when a species mostly encounters few pathogens in a limited range of habitats, resulting in reduced defense against pathogens in novel habitats (Laine, 2006; Stump et al., 2020). In extreme cases, a habitat specialist is too isolated to encounter any pathogens, leading to the loss of pathogen defense (Gibson et al., 2010). Once hosts disperse beyond native habitats, the limited diversity of R‐genes allows novel pathogens from close relatives to invade more easily (Marden et al., 2017).

Additionally, we found that the soil of local species increased the survival and growth of the conspecific seedlings, relative to the sterilized soil treatment. This suggests that mutualistic soil microbes associated with the local species facilitate the self‐recruitment and growth of conspecific seedlings. This finding is concordant with previous plant–soil feedback studies, which show that conspecific soil feedback is generally positive for temperate woody species (including oaks of eastern North America used in our study; LaManna et al., 2017). In the case of temperate oak species, soil microbes from adult trees indeed show positive feedback to conspecific seedling survival and growth, as compared to growing in heterospecific or sterile soil (Bennett et al., 2017; McCarthy‐Neumann & Ibáñez, 2012).

This positive conspecific soil feedback that we observed is likely linked to ectomycorrhizal association. Ectomycorrhizal fungi, a fungal group commonly associated with oaks, often generate positive plant–soil feedback and thus facilitate the self‐recruitment of the locally abundant species (Connell & Lowman, 1989). Consistent with our support for the host‐specificity mechanism, previous research did find host‐specific ectomycorrhizal fungi associated with different oak species (Aponte et al., 2010; Morris et al., 2008, 2009), suggesting that the mutualistic effect through fungi is determined by host identity. This specificity might explain why we observed a positive effect of local sister live soil only on local species seedlings, but a negative effect on foreign sister species. It is worth noting that in tropical ecosystems, such positive conspecific feedback is often weakened and even replaced by negative conspecific feedback (Comita et al., 2010; LaManna et al., 2017). Therefore, the roles of soil microbes in maintaining habitat restrictions of plants might be weakened or not supported for tropical species. We encourage future studies to utilize our experimental design (Figure 1) and to further compare habitat restriction through soil microbes in temperate vs. tropical plant species.

Interestingly, we found that even in sterilized soil, sister species show inherent differences in competitiveness: *Q. michauxii* and *Q. shumardii* showed higher survival compared to their sister species, *Q. alba* and *Q. acerifolia*, respectively (Figure 3). In sterilized soil, *Q. michauxii* also showed higher biomass compared to its sister species, *Q. alba* (Figure 3; Figure S3). This could be the result of seed size differences: *Q. michauxii* produces larger nuts (25–35 × 20–25 mm) as compared to *Q. alba* (15–21 × 9–18 mm) (Flora of North America, 1993). Similarly, *Q. shumardii* produces larger nuts (14–30 × 10–20 mm) than *Q. acerifolia* does (10.5–20 × 9–15 mm) (Flora of North America, 1993). It has been reported that larger acorn size is associated with higher survival and growth of oak seedlings due to the large nutrition reservoir available for resprouts and initial growth (Aizen & Patterson III, 1990; Aizen & Woodcock, 1996; Quero et al., 2007). Thus, in our study system, sister species with larger acorns have inherently higher survival rates and growth potential. In addition to acorn size, another explanation for this difference is that the water regime and soil substrate used in this experiment are more similar to the microhabitats where *Q. michauxii* and *Q. shumardii* grow in the wild. Both *Q. michauxii* and *Q. shumardii* are naturally found in wet bottomland and well‐drained soils along streams and rivers, and thus, they are more adapted to higher soil moisture (corresponding to our regular watering in the greenhouse) and higher soil nutrient level (the potting mix used for the experiment). In contrast, their sister species are both adapted to drought and upland soil. Therefore, our greenhouse conditions might have also contributed to the differences in the sterilized treatment. Nevertheless, our experiment showed that microbes from local sister live soil changed the difference in competitiveness (Figure 3): for example, *Q. michauxii* (foreign species) with larger acorns reduced its survival rates to the extent that its survival is lower than that of *Q. alba* with smaller acorns; *Q. shumardii* (local species) with larger acorns increased its survival rates and increased the gap with *Q. acerifolia*.

Interactions with soil microbes should be regarded as a partial factor contributing to species habitat restriction, but not the full explanation for why the two foreign species (*Q. michauxii* and *Q. acerifolia*) were not found beyond their habitats. Habitat restriction can be affected by a combination of other factors, including microclimatic differences, soil chemistry, and other forms of biotic interactions related to host habitats. It is possible that abiotic and biotic processes limit habitat distributions simultaneously and even synergistically (Lau et al., 2008; Rajakaruna, 2017). Indeed, our field observations and plot‐level analyses indicate that sister species rarely co‐occur within the same habitat, even though their species ranges overlap at a biogeographic scale (see Note S1, Figure S1). In an evolutionary time scale, this habitat exclusion between sister species can be first initiated by interspecific competition, and subsequently followed by local adaptation to different soil moisture levels and substrates to reduce interspecific competition. In oaks, edaphic niche partitioning is often found among closely related species and among co‐occurring species (Cavender‐Bares et al., 2004; Cavender‐Bares & Pahlich, 2009), which indicates their evolutionary lability regarding adaptation and specialization to soil factors. This adaptation to abiotic conditions can be followed by co‐evolution with specific soil microbes that optimize survival and growth in local soil conditions (Osborne et al., 2018). A genomic analysis further suggests that oaks have a high diversity of R‐genes, and thus, a potentially high capacity to co‐evolve with soil pathogens and mutualists (Plomion et al., 2018). Therefore, it is possible that the initial adaptation to soil conditions is a primary force that drives niche partitioning between sister species, while soil microbes might serve as a secondary evolutionary force that maintains niche partitioning.

Some limitations of the experiment should be recognized. One caveat of this experiment is the limited representation of genetic diversity of seed sources, since we used seeds from a small number of ex‐situ or cultivated individuals (Note S2) instead of gathering seeds representative from multiple wild populations across target species' ranges. A soil inoculum experiment that uses representative wild seeds will be needed to more accurately measure the effects of soil microbes in our study system. Second, we did not test the other direction of plant–soil interactions by introducing foreign sister's live soil to the seedlings of local sister species. Without this treatment, we cannot determine whether the habitat exclusion is symmetrical (i.e., equal strength of negative suppression from each host species) or asymmetrical. A reciprocal soil inoculum experiment will be needed to test whether the effect of soil microbes is bidirectional.

## 5 CONCLUSIONS

The role that biotic interactions play in mediating species habitat distribution is just coming to the forefront (Hargreaves et al., 2014; Katz et al., 2017; Sexton et al., 2009). Using a well‐designed soil inoculum experiment, we found that host‐specific soil microbes can contribute to habitat restriction of closely related oaks. Our finding implies that species habitat distributions are more than a simple function of abiotic constraints. Particularly, we demonstrate that considering the effects of soil microbial communities and the phylogenetic relationships among host plants will be essential to fully capture the factors determining fine‐scaled plant distributions (Benning & Moeller, 2020, 2021; Kempel et al., 2018; McCarthy‐Neumann & Ibáñez, 2012; Pither et al., 2018). We encourage future studies and native plant conservation to account for the effects of belowground biotic interactions on species habitat preferences and habitat partitioning.

## AUTHOR CONTRIBUTIONS

**Yingtong Wu:** Conceptualization (lead); data curation (lead); formal analysis (lead); funding acquisition (lead); investigation (lead); methodology (lead); project administration (lead); resources (lead); software (lead); validation (lead); visualization (lead); writing – original draft (lead); writing – review and editing (lead). **Alicia Brown:** Data curation (equal); methodology (equal). **Robert E. Ricklefs:** Conceptualization (supporting); funding acquisition (supporting); project administration (supporting); supervision (lead); writing – review and editing (equal).

## CONFLICT OF INTEREST

None declared.

## Supporting information


Appendix S1

## Data Availability

Raw data and codes used in this study are available in Dryad Data Repository (doi: https://doi.org/10.5061/dryad.fqz612jt0).
