# Supplementary material for: Host‐specific soil microbes contribute to habitat restriction of closely related oaks (Quercus spp.)
Source: Ecol Evol. 2022 Dec 12;12(12):e9614. doi: 10.1002/ece3.9614 (PMC9745265; doi:10.1002/ece3.9614)
Supplement: Supplementary file 1 — Appendix S1 [file ECE3-12-e9614-s001.docx]

# Supplementary Materials

**Title: Host-specific soil microbes** **contribute to habitat restriction of closely related oaks (*Quercus* spp.)**

**Journal: *Ecology and Evolution***

**Submission type: Research Article**

## **Note S1 Testing limited co-occurrences of sister species**

To test limited co-occurrence between sister species, we examined whether local abundance at the plot level was negatively correlated between sister species. First, we downloaded the plot-level data from the Forest Inventory and Analysis (FIA) Database (Burrill 2018). We used the “getFIA” function in the R package *rFIA* (Stanke et al. 2020) to download TREE tables from the FIA Database; theses tables document all trees whose diameter of breadth height is larger than 12.7 cm in plots of 54.53 m radius. We selected the target oak species from the dataset. Because FIA surveys were conducted in different years for different plots, we only selected the most recent survey for each plot to avoid data redundancy. The local abundance at the plot level was then transformed as log(original number of trees per plot x 1000,000) and visualized on maps (Fig. S1). Because FIA surveys do not include *Quercus acerifolia*, we obtained the occurrence records of this species from botanist Brent Baker, Arkansas Natural Heritage Commission.

To statistically test the correlation, we used generalized linear mixed model (GLMM) with a zero-inflated Poisson distribution because a high number of plots have zero abundance for one of the sister species. We used the function “glmmTMB” in R package *glmmTMB* (Magnusson et al. 2017) to perform modelling between *Q. alba* and *Q. michauxii*. We used inventory year as the random-effect intercept in the models. Indeed, we found that the local abundance of *Q. alba* and *Q. michauxii* are negatively correlated (*P* = 0.002). For the species pair of *Q. shumardii* and *Q. acerifolia*, there is no co-occurrence at the plot level between the two species, and thus a statistical test is not needed.


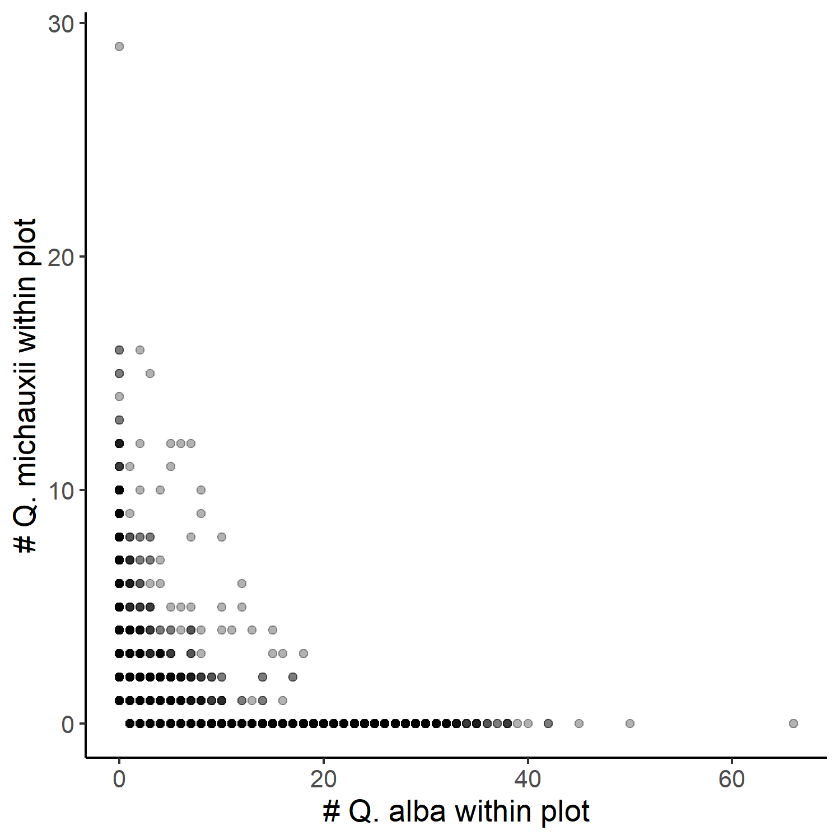


**Figure: Scatterplot showing the relationship of the local abundance within a sister-species pair of *Quercus*.** Each dot represents the number of adult trees co-occurred in a standardized Forest Inventory and Analysis (FIA) plot.

## **Note S2 Provenance information of the oak trees from which we collected acorns**

The table below shows the seed sources and information of maternal trees of the study species. The accession numbers and more info can be found at the Missouri Botanical Garden (MOBOT) Plant Finder website (<https://livingcollections.org/mobot/AccessionSearch.aspx>), or the University of Missouri—St. Louis (UMSL) TreeKeeper website (<https://univofmostl.treekeepersoftware.com/>). When accession numbers are not available, the coordinates of the trees are provided instead.

| **Species** | **Relationship** | **Number of maternal trees** | **Accession number / location** | **Provenance of maternal tree** |
| --- | --- | --- | --- | --- |
| *Q. acerifolia* | Sister species of *Q. shumardii* | 1 | 1992-1254-31 (MOBOT) | Wild; Sugarloaf Mountain, Sebastian County, AR |
| *Q. michauxii* | Sister species of *Q. alba* | 2 | 1991-3026-1 (MOBOT) | Cultivated, from Heartland Nursery, New Madrid, MO |
|  |  |  | 1969-5323-1 (MOBOT) | Unknown (Insufficient data to determine provenance) |
| *Q. shumardii* | Sister species of *Q. acerifolia* | 2 | NA / 38.48°N, 90.82°W | Wild |
|  |  |  | NA / 38.47°N, 90.82°W | Wild |
| *Q. alba* | Sister species of *Q. michauxii* | 2 | 1986-0083-1 (MOBOT) | Unknown (Insufficient data to determine provenance type) |
|  |  |  | 1281 (UMSL) | Unknown (Insufficient data to determine provenance type) |

## **Note S3 Collection of live soil from other host species at the Tyson Research Center**

In addition to our study species, the host plants (adult trees, DBH > 10 cm) at the Tyson Research Center include *Quercus rubra* (relative abundance 21.61%), *Q. velutina* (10.59%), and *Sassafras albidum* (6.55%) (see panel a below). The mean distance of an adult tree to the nearest neighbor is 3.53m (see panel b below). The data are from the most recent 2015 census based on the 20-ha ForestGEO plot at the Tyson Research Center.

*
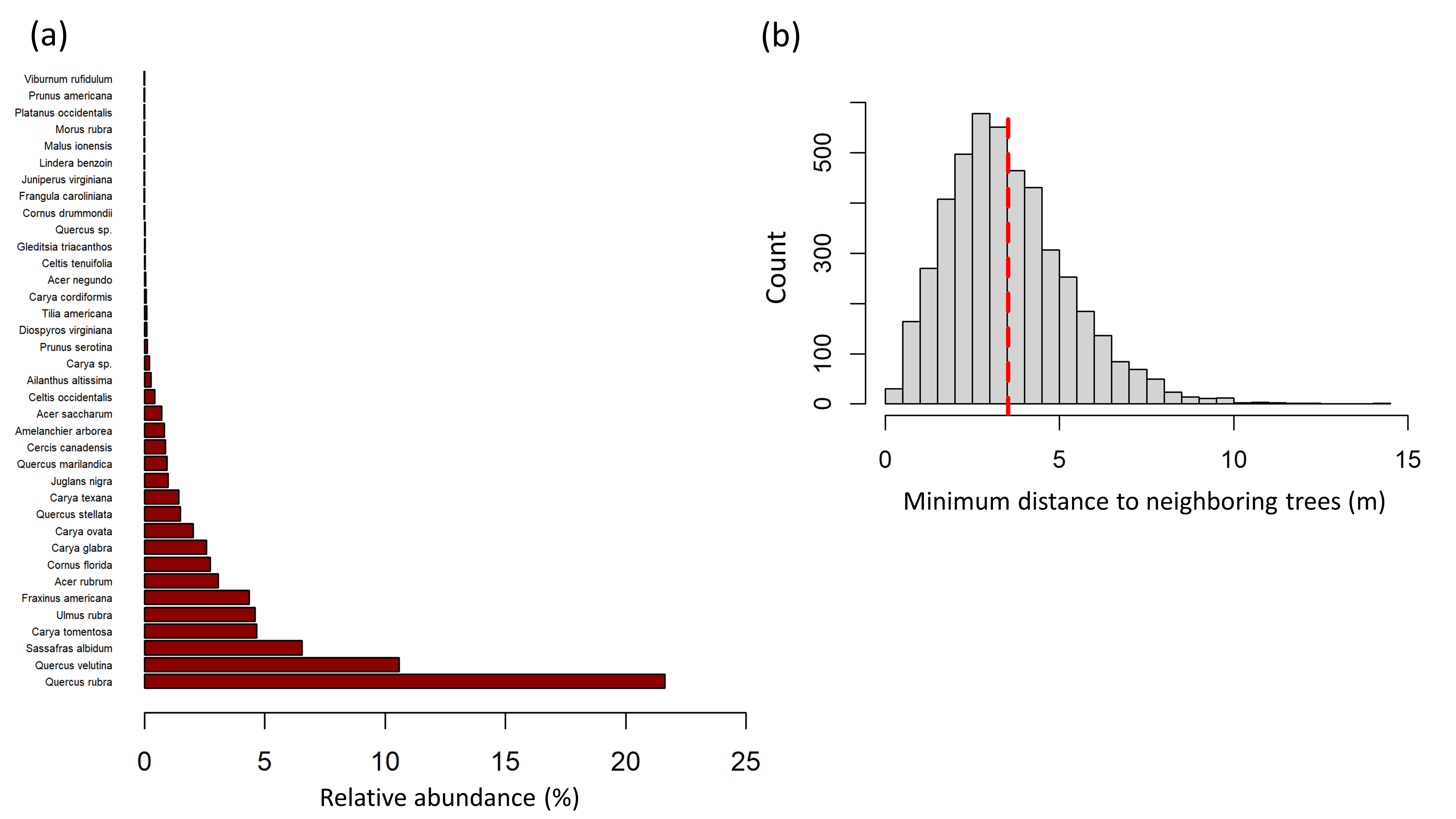
*

**Figure: The relative abundance of adult trees (DBH > 10cm) and distance to the nearest neighboring trees at the Tyson Research Center.** (a) Relative abundance of species. Trees labeled as “*Quercus* sp.” and “*Carya* sp.” have inconclusive species identities, possibly representing hybrids. (b) A histogram showing the distance to the nearest adult tree. A dashed red line represents the mean value (3.53 m).


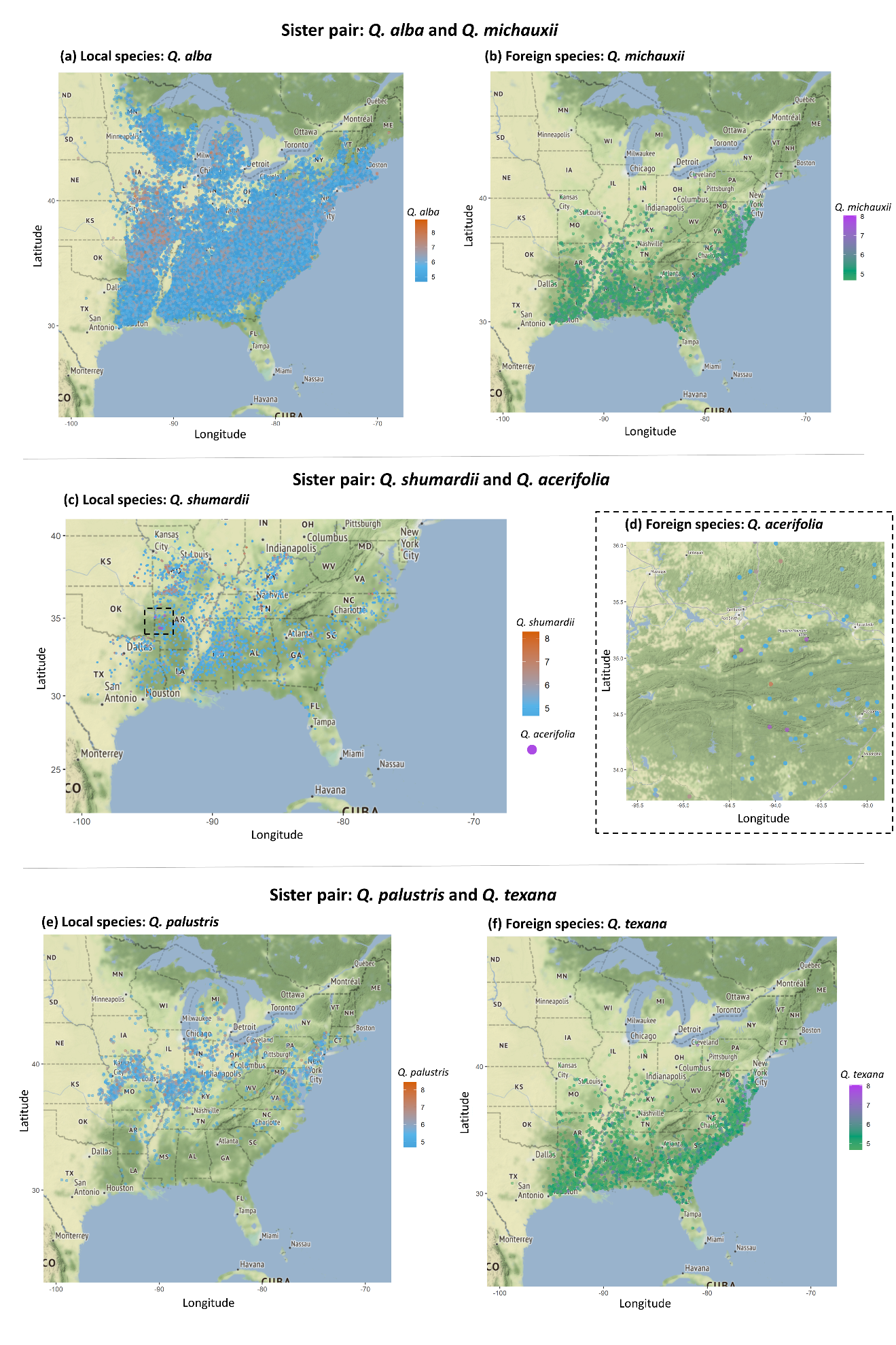


## **Figure S1 Distribution of the *Quercus* species used in this study.** Each dot represents a plot in the Forest Inventory and Assessment (FIA) Database, and the color scale represents local abundance in the unit of log(number of individuals per square meter x 1000,000). Panel (d) corresponds to the dashed square section in panel (c). Because *Quercus acerifolia* only occurs at four locations, its abundance is only represented by purple dots instead of a color gradient in panels c, d. Exact coordinates of the endangered *Q. acerifolia* should not be publicly accessible, and we used one point to represent each of the four populations for this species.


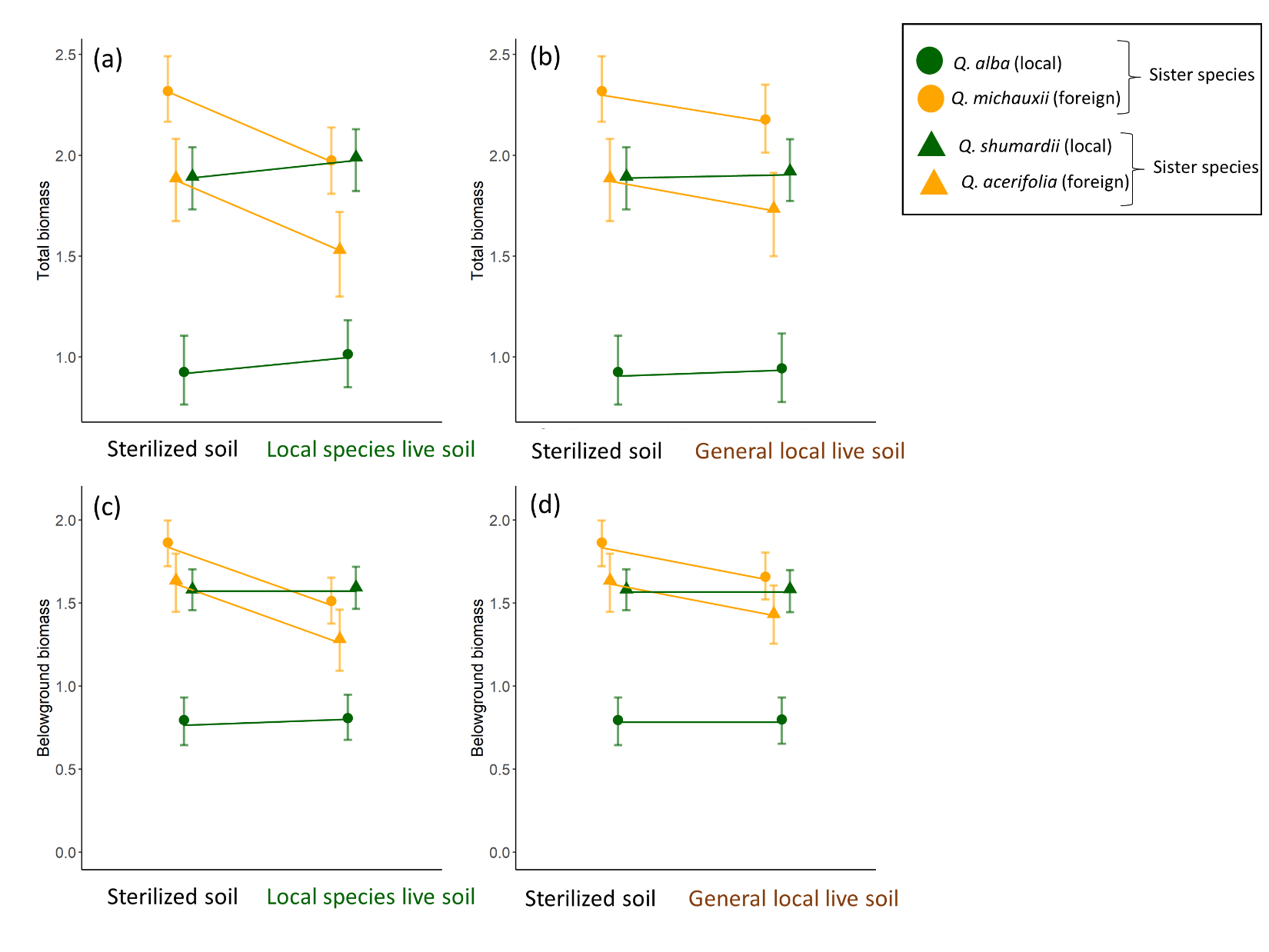


## **Figure S2 Seedling total biomass and belowground biomass of the local vs. foreign sister species in different soil treatments.** Values were derived from the best Bayesian model, using estimated marginal means. Panels (a, c) compare the total and belowground biomass of local sister (green points) when grown in sterilized soil vs. in local sister live soil, and the survival of foreign sister (yellow points) in these two treatments. Panels (b, d) compare the total and belowground biomass of local sister (green points) when grown in sterilized soil vs. in local habitat live soil that does not associate specifically with one host, and the biomass of foreign sister (yellow points) in these two treatments.


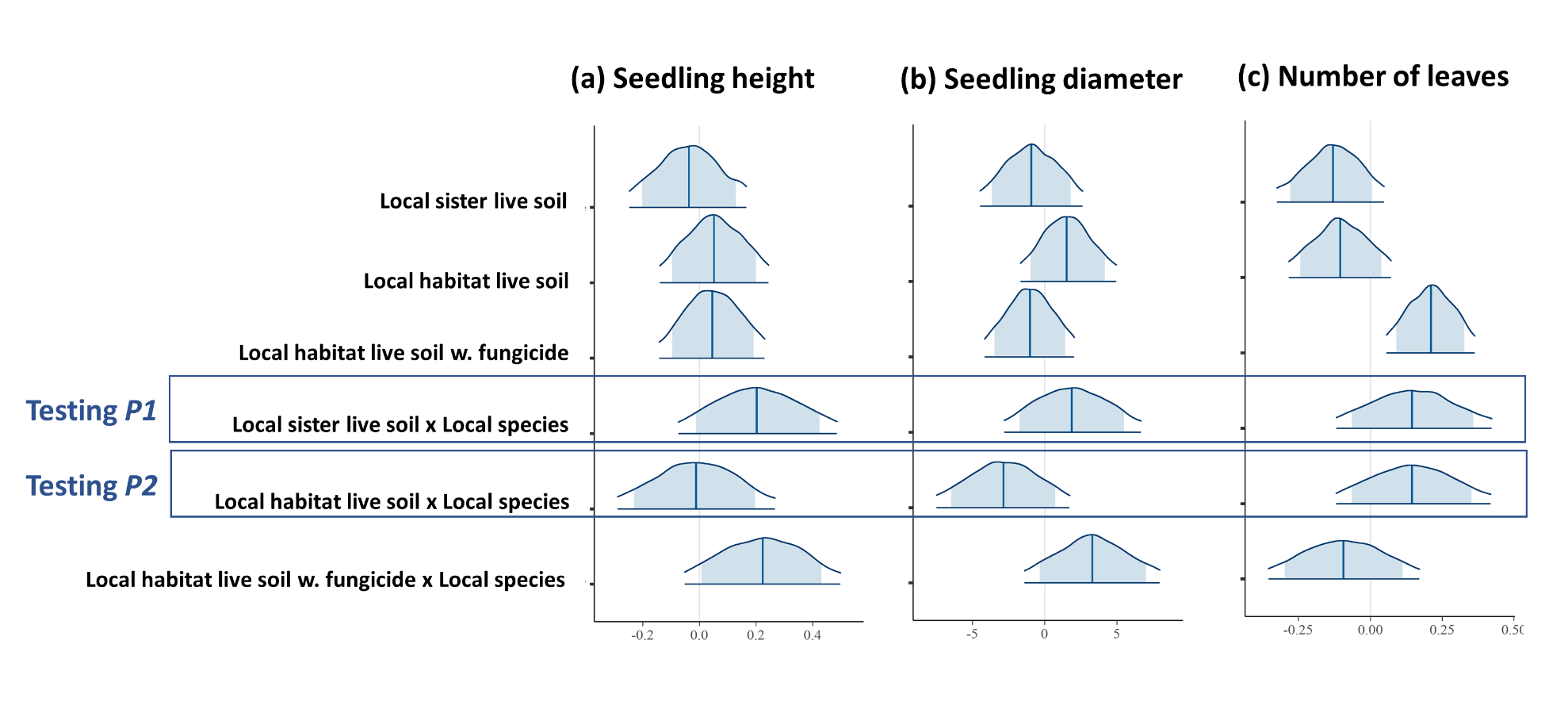


## **Figure S3 Bayesian estimates of the effects of soil treatments and host habitat origin (local species vs. foreign species) on oak seedling height, diameter, and number of leaves in a soil inoculum experiment.** Sterilized soil is used as a reference level for soil treatment, and foreign species is used as a reference level for host habitat origin. Blue vertical lines represent median estimates of the coefficients derived from the Bayesian model**s**. The truncated distribution outline represents 90% credible intervals (CIs), while the shaded-light blue region represents 80% CIs. A light-grey vertical line marks *x* = 0 in each panel. The tests for Prediction 1 (*P1*) and Prediction 2 (*P2*) are highlighted with rectangles.

## **Table S1 Summary of the best model for each response variable in the soil inoculum experiment.** The best model was identified using the “dredge” function in R package *MuMIn*, according to the corrected Akaike Information Criterion (AICc).

| **Response variable** | **Best model identified** | **Distribution** | **R function used for maximum-likelihood model** | **R function used for Bayesian model** |
| --- | --- | --- | --- | --- |
| Survival | Survival~Soil.treatment*Host.habitat.origin+Species+(1\|mother.tree) | Binomial | glmer | stan_glmer |
| Total biomass | Total.biomass ~ Seed.length+ Species + Soil.treatment*Host.habitat.origin+ (1\|mother.tree) | Normal | lmer | stan_lmer |
| Aboveground biomass | Aboveground.biomass ~ Seed.length+ Species + Soil.treatment*Host.habitat.origin + (1\|mother.tree) | Normal | lmer | stan_lmer |
| Belowground biomass | Belowgroun.biomass ~ Species.pair + Seed.length+ Species + Soil.treatment*Host.habitat.origin + (1\|mother.tree) | Normal | lmer | stan_lmer |
| Seedling height | Seedling.height ~ Seed.length+ Species + Soil.treatment*Host.habitat.origin+ (1\|mother.tree) | Normal | lmer | stan_lmer |
| Seedling diameter | Seedling.diameter ~ Seed.length+ Species + Soil.treatment*Host.habitat.origin+ (1\|mother.tree) | Normal | lmer | stan_lmer |
| Number of leaves | Number.of.leaves ~ Species.pair + Seed.length+ Species + Soil.treatment*Host.habitat.origin + (1\|mother.tree) | Poisson | glmer | stan_glmer |

## **Table S2 Model output of Bayesian generalized linear models predicting oak seedling survival in response to soil treatments, species host habitat origin, and their interactive effects in the soil inoculum experiment.** Model is fitted with binomial posterior distribution (family = “binomial”). Lower and upper boundaries mark the range of the 90% credible intervals of the Bayesian estimates. Significant effects are highlighted in bold, when the 90% credible intervals (CIs) do not include zero.

| ***Sterilized soil as reference*** | | | |
| --- | --- | --- | --- |
|  | **Variable** | **Lower boundary** | **Upper boundary** |
|  | Intercept | -0.042 | 2.322 |
| Main effects | Treatment: Local sister live soil | -2.292 | 0.106 |
|  | Treatment: Local habitat live soil | -1.577 | 0.966 |
|  | Treatment: Local habitat live soil with fungicide | -0.588 | 2.536 |
| Interactive effects | **Local sister live soil x Local sister** | **0.446** | **4.378** |
|  | Local habitat live soil x Local sister | -1.455 | 2.050 |
|  | Local habitat live soil with fungicide x Local sister | -3.258 | 0.773 |
|  |  |  |  |
| ***Local habitat live soil as reference*** | | | |
|  | **Variable** | **Lower boundary** | **Upper boundary** |
|  | Intercept | -0.282 | 2.138 |
| Main effects | Treatment: Local habitat live soil with fungicide | -0.256 | 2.690 |
|  | Treatment: Local sister live soil | -1.942 | 0.362 |
|  | Treatment: Sterilized soil | -1.035 | 1.559 |
| Interactive effects | Local habitat live soil with fungicide x Local sister | -3.370 | 0.522 |
|  | **Local sister live soil x Local sister** | **0.214** | **4.207** |
|  | Sterilized soil x Local sister | -2.032 | 1.531 |

## **Table S3** **Model output of Bayesian linear mixed-effect model predicting oak seedling biomass in response to soil treatments, species host habitat origin, and their interactive effects in the soil inoculum experiment.** Lower and upper boundaries mark the range of the 90% credible intervals of the Bayesian estimates. Significant predictors are highlighted in bold, when the 90% credible intervals (CI) do not include zero.

| ***Total biomass (sterilized soil as reference)*** | | | |
| --- | --- | --- | --- |
|  | **Variable** | **Lower boundary** | **Upper boundary** |
|  | Intercept | 1.187 | 2.468 |
| Main effects | **Treatment: Local sister live soil** | **-0.672** | **-0.009** |
|  | Treatment: Local habitat live soil | -0.454 | 0.158 |
|  | Treatment: Local habitat live soil with fungicide | -0.706 | -0.096 |
|  | **Seed length** | **0.122** | **0.581** |
| Interactive effects | Local sister live soil x Local sister | -0.009 | 0.873 |
|  | Local habitat live soil x Local sister | -0.256 | 0.612 |
|  | **Local habitat live soil with fungicide x Local sister** | **0.145** | **1.019** |
|  |  |  |  |
| ***Total biomass (Local habitat live soil as reference)*** | | | |
|  | **Variable** | **Lower boundary** | **Upper boundary** |
|  | Intercept | 1.013 | 2.375 |
| Main effects | Treatment: Local habitat live soil with fungicide | -0.550 | 0.038 |
|  | Treatment: Local sister live soil | -0.534 | 0.131 |
|  | Treatment: Sterilized soil | -0.160 | 0.469 |
|  | **Seed length** | **0.120** | **0.584** |
| Interactive effects | Local habitat live soil with fungicide x Local sister | -0.014 | 0.854 |
|  | Local sister live soil x Local sister | -0.174 | 0.716 |
|  | Sterilized soil x Local sister | -0.640 | 0.244 |
|  |  |  |  |
| ***Aboveground biomass (sterilized soil as reference)*** | | | |
|  | **Variable** | **Lower boundary** | **Upper boundary** |
|  | Intercept | 0.570 | 1.580 |
| Main effects | **Treatment: Local sister live soil** | **-0.458** | **-0.014** |
|  | Treatment: Local habitat live soil | -0.252 | 0.167 |
|  | Treatment: Local habitat live soil with fungicide | -0.358 | 0.053 |
|  | **Seed length** | **0.149** | **0.460** |
| Interactive effects | **Local sister live soil x Local sister** | **0.063** | **0.682** |
|  | Local habitat live soil x Local sister | -0.238 | 0.370 |
|  | **Local habitat live soil with fungicide x Local sister** | **0.136** | **0.741** |
|  |  |  |  |
| ***Aboveground biomass (Local habitat live soil as reference)*** | | | |
|  | **Variable** | **Lower boundary** | **Upper boundary** |
|  | Intercept | 0.536 | 1.515 |
| Main effects | Treatment: Local habitat live soil with fungicide | -0.320 | 0.104 |
|  | Treatment: Local sister live soil | -0.420 | 0.052 |
|  | Treatment: Sterilized soil | -0.171 | 0.259 |
|  | **Seed length** | **0.149** | **0.472** |
| Interactive effects | **Local habitat live soil with fungicide x Local sister** | **0.064** | **0.688** |
|  | Local sister live soil x Local sister | -0.020 | 0.610 |
|  | Sterilized soil x Local sister | -0.379 | 0.235 |
|  |  |  |  |
| ***Belowground biomass (sterilized soil as reference)*** | | | |
|  | **Variable** | **Lower boundary** | **Upper boundary** |
|  | Intercept | 1.433 | 2.315 |
| Main effects | **Treatment: Local sister live soil** | **-0.670** | **-0.029** |
|  | Treatment: Local habitat live soil | -0.504 | 0.112 |
|  | **Treatment: Local habitat live soil with fungicide** | **-0.863** | **-0.281** |
|  | **Seed length** | **0.093** | **0.526** |
| Interactive effects | Local sister live soil x Local sister | -0.056 | 0.783 |
|  | Local habitat live soil x Local sister | -0.241 | 0.623 |
|  | **Local habitat live soil with fungicide x Local sister** | **0.223** | **1.091** |
|  |  |  |  |
| ***Belowground biomass (Local habitat live soil as reference)*** | | | |
|  | **Variable** | **Lower boundary** | **Upper boundary** |
|  | Intercept | 1.251 | 2.117 |
| Main effects | **Treatment: Local habitat live soil with fungicide** | **-0.657** | **-0.072** |
|  | Treatment: Local sister live soil | -0.464 | 0.186 |
|  | Treatment: Sterilized soil | -0.092 | 0.513 |
|  | **Seed length** | **0.093** | **0.533** |
| Interactive effects | Local habitat live soil with fungicide x Local sister | -0.002 | 0.867 |
|  | Local sister live soil x Local sister | -0.312 | 0.579 |
|  | Sterilized soil x Local sister | -0.672 | 0.203 |

## **Table S4 Models output of Bayesian models predicting oak seedling height, diameter, and number of leaves in the soil inoculum experiment.** Seedling height and seedling diameter are fitted with a linear mixed model. While the number of leaves is fitted with a generalized linear model with a Poisson posterior distribution. Lower and upper boundaries mark the range of the 90% credible intervals of the Bayesian estimates. Significant effects are highlighted in bold, when the 90% credible intervals (CIs) do not include zero.

| ***Seedling height (sterilized soil as reference)*** | | | |
| --- | --- | --- | --- |
|  | **Variable** | **Lower boundary** | **Upper boundary** |
|  | Intercept | 2.519 | 3.509 |
| Main effects | Treatment: Local sister live soil | -0.246 | 0.166 |
|  | Treatment: Local habitat live soil | -0.140 | 0.245 |
|  | Treatment: Local habitat live soil with fungicide | -0.142 | 0.231 |
|  | **Seed length** | **0.027** | **0.320** |
| Interactive effects | Local sister live soil x Local sister | -0.073 | 0.486 |
|  | Local habitat live soil x Local sister | -0.288 | 0.268 |
|  | Local habitat live soil with fungicide x Local sister | -0.051 | 0.498 |
|  |  |  |  |
| ***Seedling height (Local habitat live soil as reference)*** | | | |
|  | **Variable** | **Lower boundary** | **Upper boundary** |
|  | Intercept | 2.589 | 3.539 |
| Main effects | Treatment: Local habitat live soil with fungicide | -0.196 | 0.180 |
|  | Treatment: Local sister live soil | -0.306 | 0.128 |
|  | Treatment: Sterilized soil | -0.249 | 0.143 |
|  | **Seed length** | **0.025** | **0.310** |
| Interactive effects | Local habitat live soil with fungicide x Local sister | -0.032 | 0.516 |
|  | Local sister live soil x Local sister | -0.070 | 0.496 |
|  | Sterilized soil x Local sister | -0.269 | 0.286 |
|  |  |  |  |
| ***Seedling diameter biomass (sterilized soil as reference)*** | | | |
|  | **Variable** | **Lower boundary** | **Upper boundary** |
|  | Intercept | 15.988 | 29.381 |
| Main effects | Treatment: Local sister live soil | -4.477 | 2.623 |
|  | Treatment: Local habitat live soil | -1.680 | 4.962 |
|  | Treatment: Local habitat live soil with fungicide | -4.152 | 2.052 |
|  | **Seed length** | **1.393** | **6.336** |
| Interactive effects | Local sister live soil x Local sister | -2.790 | 6.668 |
|  | Local habitat live soil x Local sister | -7.490 | 1.720 |
|  | Local habitat live soil with fungicide x Local sister | -1.397 | 7.967 |
|  |  |  |  |
| ***Seedling diameter (Local habitat live soil as reference)*** | | | |
|  | **Variable** | **Lower boundary** | **Upper boundary** |
|  | Intercept | 17.603 | 30.894 |
| Main effects | Treatment: Local habitat live soil with fungicide | -5.788 | 0.644 |
|  | Treatment: Local sister live soil | -6.145 | 1.155 |
|  | Treatment: Sterilized soil | -4.868 | 1.776 |
|  | **Seed length** | **1.467** | **6.296** |
| Interactive effects | **Local habitat live soil with fungicide x Local sister** | **1.251** | **10.974** |
|  | Local sister live soil x Local sister | -0.217 | 9.611 |
|  | Sterilized soil x Local sister | -2.002 | 7.613 |
|  |  |  |  |
| ***Number of leaves (sterilized soil as reference)*** | | | |
|  | **Variable** | **Lower boundary** | **Upper boundary** |
|  | Intercept | 2.202 | 2.897 |
| Main effects | Treatment: Local sister live soil | -0.324 | 0.048 |
|  | Treatment: Local habitat live soil | -0.283 | 0.072 |
|  | **Treatment: Local habitat live soil with fungicide** | **0.056** | **0.364** |
|  | **Seed length** | **0.008** | **0.276** |
| Interactive effects | Local sister live soil x Local sister | -0.117 | 0.422 |
|  | Local habitat live soil x Local sister | -0.119 | 0.419 |
|  | Local habitat live soil with fungicide x Local sister | -0.354 | 0.171 |
|  |  |  |  |
| ***Number of leaves (Local habitat live soil as reference)*** | | | |
|  | **Variable** | **Lower boundary** | **Upper boundary** |
|  | Intercept | 2.100 | 2.769 |
| Main effects | **Treatment: Local habitat live soil with fungicide** | **0.144** | **0.477** |
|  | Treatment: Local sister live soil | -0.224 | 0.163 |
|  | Treatment: Sterilized soil | -0.077 | 0.281 |
|  | **Seed length** | **0.010** | **0.281** |
| Interactive effects | Local habitat live soil with fungicide x Local sister | -0.498 | 0.034 |
|  | Local sister live soil x Local sister | -0.256 | 0.281 |
|  | Sterilized soil x Local sister | -0.414 | 0.129 |

## **References**

Burrill, E. A. W., Andrea M.; Turner, Jeffery A.; Pugh, Scott A.; Menlove, James; Christiansen, Glenn; Conkling, Barbara L.; David, Winnie. 2018. U.S. Department of Agriculture, Forest Service.

Magnusson, A., H. Skaug, A. Nielsen, C. Berg, K. Kristensen, M. Maechler, K. van Bentham, B. Bolker, M. Brooks, and M. M. Brooks. 2017. Package ‘glmmTMB’. R Package Version 0.2. 0.

Stanke, H., A. O. Finley, A. S. Weed, B. F. Walters, and G. M. Domke. 2020. rFIA: An R package for estimation of forest attributes with the US Forest Inventory and Analysis database. Environmental Modelling & Software **127**:104664.
